# Colorectal cancer screening: the time to act is now

**DOI:** 10.1186/s12916-015-0498-x

**Published:** 2015-10-13

**Authors:** Hermann Brenner, Christian Stock, Michael Hoffmeister

**Affiliations:** Division of Clinical Epidemiology and Aging Research, German Cancer Research Center (DKFZ), INF 581, 69120 Heidelberg, Germany; Division of Preventive Oncology, German Cancer Research Center (DKFZ) and National Center for Tumor Diseases (NCT), INF 460, 69120 Heidelberg, Germany; German Cancer Consortium (DKTK), German Cancer Research Center (DKFZ), INF 280, 69120 Heidelberg, Germany; Institute of Medical Biometry and Informatics, University of Heidelberg, INF 305, 69120 Heidelberg, Germany

**Keywords:** Colorectal cancer, Colonoscopy, Faecal immunochemical tests, Faecal occult blood tests, Flexible sigmoidoscopy, Incidence, Microsimulation models, Mortality, Screening

## Abstract

**Background:**

Colorectal cancer (CRC) is the third most common cancer and the fourth most common cause of cancer deaths globally. However, there is overwhelming evidence that a large proportion of CRC cases and deaths could be prevented by screening. Nevertheless, CRC screening programmes are offered in a minority of countries only and often suffer from low adherence.

**Discussion:**

Factors potentially accounting for hesitant implementation of and low adherence to CRC screening may include a lower attention in the public and the media than for other cancers and the fairly long follow-up time needed to fully disclose screening effects on CRC incidence and mortality. The latter results from the very slow development of most CRCs through the adenoma-carcinoma sequence, and it challenges the predominant or even exclusive reliance on evidence from randomized controlled trials in policy decisions on screening offers. Additional key elements of future research should include (1) studies evaluating diagnostic performance of novel biomarkers for non-invasive or minimally invasive CRC screening in true screening settings, (2) modelling studies evaluating expected short- and long-term impact, effectiveness, and cost-effectiveness of various screening options, and (3) timely and close monitoring of process quality and outcomes of existing and planned CRC screening programmes. Most importantly, however, translation of the vast existing evidence on CRC screening into actual screening programmes with the best possible levels of adherence needs to be fostered. This can be best achieved in the context of organized programmes. Depending on available infrastructure and resources, epidemiological patterns, population preferences, and costs, different screening offers might be preferred. According to current evidence, colonoscopy, flexible sigmoidoscopy, and faecal occult blood tests (preferably faecal immunochemical tests) are prime candidates for effective and cost-effective screening options, and microsimulation models should help to tailor their implementation.

**Summary:**

The strong evidence for the large potential of CRC screening in reducing the burden of CRC calls for timely implementation of organized screening programmes where they are not in place yet, and for continuous improvement of existing ones. This should be considered an obligation that is not to be postponed: the time to act is now.

## Background

Colorectal cancer (CRC) is the third most common cancer and the fourth most common cause of cancer deaths globally, accounting for approximately 1.4 million new cases and 700,000 deaths every year [1]. Incidence is particularly high in developed countries; in Europe, CRC is the second most common cancer. With approximately 450,000 new cases per year, case numbers are only slightly lower than those of breast cancer patients. There is meanwhile overwhelming evidence from both randomized controlled trials (RCTs) and epidemiological studies that a large proportion of CRC cases and deaths could be prevented by screening with early detection and removal of colorectal adenomas or early stage CRC [2–4]. There is also overwhelming evidence that CRC screening by either faecal occult blood test (FOBT), flexible sigmoidoscopy, or colonoscopy is both effective and cost-effective, even though uncertainty remains about which of the screening options would be the most cost-effective one [5]. Nevertheless, CRC screening programmes continue to be offered in a minority of countries only [6], and where they are offered there is often much room for enhancing screening offers and adherence. The aim of this article is to review potential reasons for this major translational gap, and to discuss implications for further research and practice.

## Discussion

### Barriers hindering or delaying implementation of and adherence to CRC screening

Several factors may have accounted for the reluctance to implement (at the national, regional, or health system level) and to adhere (at the individual level) to effective CRC screening programmes. First, despite its frequency and public health importance, CRC is a cancer that might attract less attention than other cancers in the public and the media. With a median age close to 70 years in many high income countries, it mainly affects older adults, and the organ affected may be less popular for media campaigns and other public relations activities than other organs. Second, until recently, apart from FOBT screening [2], evidence on the effectiveness of CRC screening has not been available from long-term RCTs [7–10], and no such evidence will be available for screening colonoscopy, probably the most effective screening examination, for many years to come. Third, scientists are often very focused on dissemination of their results in the scientific community, with less attention to disseminating practically relevant findings, such as the available convincing evidence on effectiveness of CRC screening, to the public and health policymakers or to fostering translation into applied measures of prevention. Fourth, politicians, healthcare stakeholders, and healthcare providers may tend to focus on measures that pay off in the short run, whereas most of the major benefits of CRC screening in terms of prevented CRC cases and deaths and of monetary savings from prevented treatment costs are to be expected in the long run only. Finally, necessary resources and infrastructure, e.g. for high quality colonoscopy and for monitoring of programme performance, continue to be limited in many countries.

### Specific chances and challenges resulting from the natural history of CRC

A specific characteristic of CRC is the very slow development of most cases through the adenoma-carcinoma sequence, which often takes decades. For example, average annual transition rates from advanced adenomas to preclinical CRC and from preclinical CRC to clinically manifest CRC have been estimated in the order of <5 % and 20 %, which translates to mean sojourn times of advanced adenomas and preclinical CRC in the order of >20 and 5 years, respectively [11, 12].

This slow development opens specific benefits such as retardation of clinical manifestation of the disease into older ages and a broad time window for detecting and removing adenomas and preclinical CRC. On the other hand, this characteristic also results in specific challenges: first, it will take several years if not decades until the full effects of screening can be demonstrated by RCTs or prospective cohort studies. For example, despite RCTs on CRC screening by FOBT having been initiated since the 1970s, full disclosure of screening effects is ongoing in the second decade of the 21st century [3, 13]. In the RCTs on screening by flexible sigmoidoscopy, the reduction of CRC incidence and mortality only became manifest after 10 or more years of follow-up (typically 15+ years after start of recruitment) [4], whereas an earlier interim analysis of one of the trials conducted after 7 years of follow-up had essentially yielded negative results [14].

Epidemiological studies and indirect evidence from sigmoidoscopy trials suggest that still substantially larger effects may be achieved by screening colonoscopy. However, the only RCT designed to assess reduction of CRC incidence and mortality by screening colonoscopy completed recruitment only recently [15], and main results after 15 years of follow-up will only become available around 2030. In an era of widespread use of colonoscopy for diagnostic purposes that takes place in many Western countries and has similar protective effects as screening colonoscopy (through detection and removal of adenomas and preclinical CRC), substantial proportions of the control group of a colonoscopy screening trial can be expected to have a colonoscopy during follow-up. Such “contamination” was already a major issue in the earlier flexible sigmoidoscopy trials [9]. If not addressed in the analyses, major contamination in the control group will lead to strong underestimation of effects in screening trials [16]. Thus, while the principle effectiveness of screening colonoscopy in reducing CRC mortality is extremely likely to be formally established by RCT evidence, the estimated magnitude of the risk reduction will be at least difficult to interpret and to generalize. Furthermore, novel biomarker tests, endoscopic technology, and training are developing rapidly. Hence, RCT results of screening colonoscopy that pertain to colonoscopy technology and training standards in 2010 will be highly interesting but might be dispensable when main results will become available around 2030.

Likewise, since initiation of the large FOBT trials, which used guaiac-based FOBTs (gFOBTs), substantially improved FOBTs have been developed. In particular, fecal immunochemical tests (FITs) have been shown to achieve substantially higher sensitivity at comparable specificity for detecting colorectal adenomas and preclinical CRC [17, 18]. Under these circumstances, *de novo* initiation of RCTs demonstrating effectiveness or superiority of FITs and other novel biomarker tests in CRC screening that would take decades to complete would not be a reasonable option.

### Implications for research and practice

Notwithstanding the undoubted importance of RCTs in demonstrating the principle effectiveness of medical interventions in general and screening in particular, the examples given above challenge the predominant or even exclusive reliance on evidence from RCTs as a basis for further progress in CRC screening. A complementary differentiated research agenda is required that should include, as a guideline, the following key elements:

- *Studies evaluating the diagnostic performance of novel biomarkers for non-invasive or minimally invasive early detection of colorectal adenomas and preclinical CRC, such as blood-, stool-, or urine-based biomarkers.* Ideally, such studies should be conducted in a true screening setting among the target population of CRC screening, and include as reference a diagnostic gold standard and established non-invasive tests, such as gFOBT and FIT, for comparison. Examples include studies conducted among participants of screening colonoscopy with biospecimen collection prior to colonoscopy (e.g. [19, 20]).
- *Modelling studies evaluating the expected short- and long-term impact, effectiveness, and cost-effectiveness of various screening options in specific target populations for screening.* Microsimulation models based on the natural history of CRC development are a particularly useful approach in this context [21, 22]. Apart from modelling overall effectiveness and cost-effectiveness of various screening methods, such approaches allow for comparative evaluation of specific design options of screening programmes, such as the start and end of screening offers at various ages, various screening intervals and follow-up schemes for surveillance after detection of colorectal adenomas, or various risk-adapted screening strategies based on a priori risk stratification. In an era of rapidly increasing cancer treatment costs, demonstration of cost-effectiveness or even cost-savings of CRC screening strategies could be particularly helpful for closing the translational gap between scientific evidence and practice of CRC screening [5].
- *Timely and close monitoring of process quality and outcomes of existing and newly introduced CRC screening programmes.* Guidelines for quality assurance have been worked out in great detail [23]. Key components include monitoring of adherence to screening and surveillance offers, positivity rates, and diagnostic performance of screening tests, follow-up, and management of positive results, stage-specific detection rates, and outcomes of colorectal adenomas and preclinical CRC, but also potential complications associated with screening-induced diagnostic measures such as colonoscopy (e.g. [24–26]).
- *Timely and close monitoring of sex- and age-specific CRC incidence and mortality in the target population of screening based on data from population-based cancer registries and mortality statistics* (e.g. [27, 28])*.* Both time trend analyses assessing trends prior to and after implementation of screening programmes in specific populations as well as comparative analyses of CRC incidence and mortality between populations with differential screening coverage are of particular interest in this context, along with studies with direct linkage of screening and cancer registries [29].

Most importantly, however, it is time to foster translation of the already available overwhelming evidence on the large potential of CRC screening into practice. This not only applies to the large number of high incidence countries where still no CRC screening programme is in place and introduction of CRC screening should have a high public health priority. There is also large potential to substantially increase the impact of CRC screening and further substantially reduce the burden of CRC incidence and mortality by enhancing adherence to screening offers. For example, model calculations have shown that most of the current CRC deaths in the United States are attributable to non-screening [30], despite substantially higher screening (especially endoscopic screening) coverage than in other countries. It has furthermore been estimated that increasing screening rates in the United States from approximately 58 % in 2013 to 80 % in 2018 would result in a reduction of CRC incidence and mortality by 22 % and 30 %, respectively, from 2013 to 2030. These reductions would amount to a total of 277,000 averted new cancers and 203,000 averted CRC deaths from 2013 through 2030 [31]. Even substantially larger effects could be achieved in countries with much lower adherence rates, such as Germany, where screening colonoscopy has been offered since the end of 2002. Although this offer was used by only 20–25 % of those eligible within the initial 10 years after its introduction, it was estimated to have prevented approximately 180,000 new cases of CRC in the long run [32]. Experience from various countries shows that high adherence rates can be best achieved by offer of screening in the context of organized screening programmes with personal invitation, follow-up of invitees, and comprehensive concepts for quality assurance and programme evaluation.

Depending on availability of health care infrastructure and resources, epidemiological patterns, population preferences, and costs, different screening options might be preferred and offered in various countries. According to evidence available to date, colonoscopy, flexible sigmoidoscopy, and FOBTs would be prime candidates for effective and cost-effective screening options which might be offered as alternative or complementary screening offers, notwithstanding the lack of RCT results for colonoscopy which will not be available for many years. Regarding FOBTs, there is convincing evidence that quality-assured use of FIT should be preferred over the use of gFOBT [33], despite restriction of direct evidence from RCTs for the latter. Apart from the choice of specific screening offers, numerous decisions have to be made, such as definition of the target population for screening, screening and surveillance intervals, and potential risk stratification in screening. Microsimulation models appear to be the most promising approach to make such decisions as evidence-based as possible and should be more widely used in this context. They might also be particularly helpful to the timely evaluation of the potential role of emerging novel non- or minimally-invasive screening tests or imaging technologies that might enhance the spectrum of effective and cost-effective screening options in the future.

## Summary

The available evidence strongly suggests that there is a large but widely underused potential for CRC screening in reducing the burden of CRC incidence and mortality. It calls for timely implementation of organized screening programmes where they are not in place yet, and for continuous improvement of existing offers where such programmes exist. This should be considered an obligation that is not to be postponed: the time to act is now.

## Abbreviations

CRC: Colorectal cancer
FIT: Faecal immunochemical test
FOBT: Faecal occult blood test
gFOBT: Guaiac-based faecal occult blood test
RCT: Randomized controlled trial

## References

[1] Ferlay J, Soerjomataram I, Ervik M, Dikshit R, Eser S, Mathers C, et al. GLOBOCAN 2012 v1.0, Cancer Incidence and Mortality Worldwide: IARC CancerBase No. 11. Lyon, France: International Agency for Research on Cancer; 2013. http://globocan.iarc.fr. Accessed 17 August 2015.

[2] Hewitson P, Glasziou P, Watson E, Towler B, Irwig L (2008). Cochrane systematic review of colorectal cancer screening using the fecal occult blood test (hemoccult): an update. Am J Gastroenterol.

[3] Shaukat A, Mongin SJ, Geisser MS, Lederle FA, Bond JH, Mandel JS (2013). Long-term mortality after screening for colorectal cancer. N Engl J Med.

[4] Brenner H, Stock C, Hoffmeister M (2014). Effect of screening sigmoidoscopy and screening colonoscopy on colorectal cancer incidence and mortality: systematic review and meta-analysis of randomised controlled trials and observational studies. BMJ.

[5] Lansdorp-Vogelaar I, Knudsen A, Brenner H (2011). Cost-effectiveness of colorectal cancer screening. Epidemiol Rev.

[6] Schreuders EH, Ruco A, Rabeneck L, Schoen RE, Sung JJ, Young GP (2015). Colorectal cancer screening: a global overview of existing programmes. Gut.

[7] Atkin WS, Edwards R, Kralj-Hans I, Wooldrage K, Hart AR, Northover JMA (2010). Once-only flexible sigmoidoscopy screening in prevention of colorectal cancer: a multicentre randomised controlled trial. Lancet.

[8] Segnan N, Armaroli P, Bonelli L, Risio M, Sciallero S, Zappa M (2011). Once-only sigmoidoscopy in colorectal cancer screening: follow-up findings of the Italian Randomized Controlled Trial – SCORE. J Natl Cancer Inst.

[9] Schoen RE, Pinsky PF, Weissfeld JL, Yokochi LA, Church T, Laiyemo AO (2012). Colorectal-cancer incidence and mortality with screening flexible sigmoidoscopy. N Engl J Med.

[10] Holme Ø, Løberg M, Kalager M, Bretthauer M, Hernan MA, Aas E (2014). Effect of flexible sigmoidoscopy screening on colorectal cancer incidence and mortality. JAMA.

[11] Brenner H, Altenhofen L, Stock C, Hoffmeister M (2013). Natural history of colorectal adenomas: Birth cohort analysis among 3.6 million participants of screening colonoscopy. Cancer Epidemiol Biomarkers Prev.

[12] Brenner H, Altenhofen L, Katalinic A, Lansdorp-Vogelaar I, Hoffmeister M (2011). Sojourn time of preclinical colorectal cancer by sex and age: estimates from the German national screening colonoscopy database. Am J Epidemiol.

[13] Scholefield JH, Moss SM, Mangham CM, Whynes DK, Hardcastle JD (2012). Nottingham trial of faecal occult blood testing for colorectal cancer: a 20-year follow-up. Gut.

[14] Hoff G, Grotmol T, Skovlund E, Bretthauer M, for the Norwegian Colorectal Cancer Prevention Study Group (2009). Risk of colorectal cancer seven years after flexible sigmoidoscopy screening: randomised controlled trial. BMJ.

[15] Kaminski MF, Bretthauer M, Zauber AG, Kuipers EJ, Adami H-O, van Ballegooijen M (2012). The NordICC Study: Rationale and design of a randomized trial on colonoscopy screening for colorectal cancer. Endoscopy.

[16] Brenner H, Stock C, Hoffmeister M (2013). In the era of widespread endoscopy use randomized trials may strongly underestimate effects of colorectal cancer screening. J Clin Epidemiol.

[17] Brenner H, Tao S (2013). Superior diagnostic performance of fecal immunochemical tests for hemoglobin in a head-to-head comparison with guaiac based fecal occult blood test among 2235 participants of screening colonoscopy. Eur J Cancer.

[18] Lee JK, Liles EG, Bent S, Levin TR, Corley DA (2014). Accuracy of fecal immunochemical tests for colorectal cancer: systematic review and meta-analysis. Ann Intern Med.

[19] Imperiale TF, Ransohoff DF, Itzkowitz SH, Levin TR, Lavin P, Lidgard GP (2014). Multitarget stool DNA testing for colorectal-cancer screening. N Engl J Med.

[20] Chen H, Zucknick M, Werner S, Brenner H (2015). Head-to head comparison and evaluation of 92 plasma protein biomarkers for early detection of colorectal cancer in a true screening setting. Clin Cancer Res.

[21] Habbema JD, Wilt TJ, Etzioni R, Nelson HD, Schechter CB, Lawrence WF (2014). Models in the development of clinical practice guidelines. Ann Intern Med.

[22] van Hees F, Zauber AG, van Veldhuizen H, Heijnen ML, Penning C, de Koning HJ (2015). The value of models in informing resource allocation in colorectal cancer screening: the case of the Netherlands. Gut.

[23] European Colorectal Cancer Screening Guidelines Working Group (2013). European guidelines for quality assurance in colorectal cancer screening and diagnosis: overview and introduction to the full supplement publication. Endoscopy.

[24] Lo SH, Halloran S, Snowball J, Seaman H, Wardle J, von Wagner C (2015). Colorectal cancer screening uptake over three biennial invitation rounds in the English bowel cancer screening programme. Gut.

[25] Stegeman I, van Doorn SC, Mundt MW, Mallant-Hent RC, Bongers E, Elferink MA (2015). Participation, yield, and interval carcinomas in three rounds of biennial FIT-based colorectal cancer screening. Cancer Epidemiol.

[26] Pox CP, Altenhofen L, Brenner H, Theilmeier A, von Stillfried D, Schmiegel W (2012). Efficacy of a nationwide screening colonoscopy program for colorectal cancer. Gastroenterology.

[27] McClements PL, Madurasinghe V, Thomson CS, Fraser CG, Carey FA, Steele RJ (2012). Impact of the UK colorectal cancer screening pilot studies on incidence, stage distribution and mortality trends. Cancer Epidemiol.

[28] Zorzi M, Fedeli U, Schievano E, Bovo E, Guzzinati S, Baracco S (2015). Impact on colorectal cancer mortality of screening programmes based on faecal immunochemical test. Gut.

[29] Anttila A, Lönnberg S, Ponti A, Suonio E, Villain P, Coebergh JW (2015). Towards better implementation of cancer screening in Europe through improved monitoring and evaluation and greater engagement of cancer registries. Eur J Cancer.

[30] Meester RG, Doubeni CA, Lansdorp-Vogelaar I, Goede SL, Levin TR, Quinn VP (2015). Colorectal cancer deaths attributable to nonuse of screening in the United States. Ann Epidemiol.

[31] Meester RG, Doubeni CA, Zauber AG, Goede SL, Levin TR, Corley DA (2015). Public health impact of achieving 80 % colorectal cancer screening rates in the United States. Cancer.

[32] Brenner H, Altenhofen L, Stock C, Hoffmeister M (2015). Prevention, early detection, and overdiagnosis of colorectal cancer within 10 years of screening colonoscopy in Germany. Clin Gastroenterol Hepatol.

[33] Tinmouth J, Lansdorp-Vogelaar I, Allison JE (2015). Faecal immunochemical tests versus guaiac faecal occult blood tests: what clinicians and colorectal cancer screening programme organisers need to know. Gut.

